# Predictive biomarkers with isatuximab plus pomalidomide and dexamethasone in relapsed/refractory multiple myeloma

**DOI:** 10.1038/s41408-021-00438-y

**Published:** 2021-03-12

**Authors:** Paul G. Richardson, Thierry Facon, William I. Bensinger, Xavier Leleu, Frank Campana, Sandrine Macé, Marielle Chiron, Helgi van de Velde, Joseph Mikhael

**Affiliations:** 1 Medical Oncology, Dana-Farber Cancer Institute, Harvard Medical School, Boston, MA USA; 2 Department of Haematology, Lille University Hospital, Lille, France; 3 Myeloma & Transplant Program, Swedish Cancer Institute, Seattle, WA USA; 4 Service d’Hématologie et Thérapie Cellulaire, Hôpital de La Milétrie, Poitiers Cedex, France; 5 Sanofi-Genzyme, Cambridge, MA USA; 6 Sanofi R&D, Vitry-sur-Seine, France; 7 Translational Genomics Research Institute (TGen), City of Hope Cancer Center, Phoenix, AZ USA; 8 Present Address: Takeda Pharmaceuticals, Cambridge, MA USA

**Keywords:** Medical research, Diseases

Dear Editor,

Multiple myeloma (MM) is the second most common hematologic disease worldwide^1^. CD38 is a type II transmembrane glycoprotein that is highly expressed on MM cells, and functions both as a receptor and as a multifunctional ectoenzyme^2^. CD38 monoclonal antibodies (mAbs) exhibit anti-MM activity via multiple mechanisms of action and are being evaluated in all stages of therapy. However, not all patients respond to CD38 mAb therapy; variability in response may be explained by both host-related and tumor-related factors^3^. The identification of biomarkers with predictive value for response could help to optimize and personalize the treatment of patients with relapsed/refractory multiple myeloma (RRMM). At present, CD38 receptor density (RD) on MM cells is the only biomarker with an association with clinical response to CD38 antibody therapies^4^.

Isatuximab, an IgG-kappa anti-CD38 mAb is effective as a single agent and is well tolerated in patients with RRMM^5,6^. It targets tumor cells via multiple mechanisms including Fc-dependent immune effector mechanisms such as antibody-dependent cellular cytotoxicity (ADCC), antibody-dependent cellular phagocytosis, and complement-dependent cytotoxicity, as well as direct apoptosis^7^. Besides initial anti-MM activity through antibody-dependent mechanisms, isatuximab also has long-term immunomodulatory effects through decrease in T regulatory (Treg) cells, increase in T-cell clonality, and induction of myeloma-specific immunity^8,9^.

Data were derived from patients enrolled in two clinical studies that have been previously described^6,10^. Study 1 (NCT02283775) was a phase-1b dose-escalation study of isatuximab in combination with pomalidomide and dexamethasone (Isa-Pd) in patients with RRMM^6^; Study 2 (ICARIA-MM study; NCT02990338) was a randomized, active-controlled, phase-3 study evaluating Isa-Pd vs. Pd^10^. In this exploratory analysis, we investigated whether baseline biomarkers including CD38 RD on bone marrow plasma cells, Fc immunoglobulin receptor (*FCGR3A*) genotype, bone marrow, and peripheral blood immunophenotypes have predictive value for treatment benefit to Isa-Pd in Studies 1 and 2.

Biomarker analyses conducted for each study are summarized in Fig. 1A. Baseline peripheral blood samples were taken prior to first treatment in both studies. In addition, a bone marrow plasma cell sample was taken during screening in Study 1. CD38 RD in bone marrow plasma cells and immune cell markers in blood and bone marrow samples were analyzed by multiparametric flow cytometry. Immune cell populations including B cells (CD19^+^ B cell), T cells (CD3^+^ T cell, CD4^+^ T cell, Tregs), and NK cells (CD56^+^ bright CD16^+^ low subset and CD56^+^ dim CD16^+^ bright subset) were characterized. The proportion of cells positive for a given marker or set of markers was correlated with response to isatuximab combination treatment. In addition, blood samples from both studies were analyzed for germline *FCGR3A* genotyping (V158 high-affinity and F158 low-affinity alleles). Biomarker levels were correlated with treatment response, defined as at least partial response according to International Myeloma Working Group criteria. Single-nucleotide substitution at amino acid position 158 in the *FCGR3A* gene (known as *FCGR3A*-V158F polymorphism) generates allotypes with different binding affinities of NK cells to tumor-bound IgG antibody. The binding of NK cells (via ligation of their low-affinity Fc receptor [CD16a] to an IgG antibody) is enhanced by the presence of a valine at position 158 (V/V or V/F) as compared with homozygous phenylalanine genotype (F/F), thereby leading to a higher level of NK-cell-mediated ADCC^11^. Patients were categorized as responders (patients with a best overall response of at least partial response) or non-responders.

**Fig. 1:**
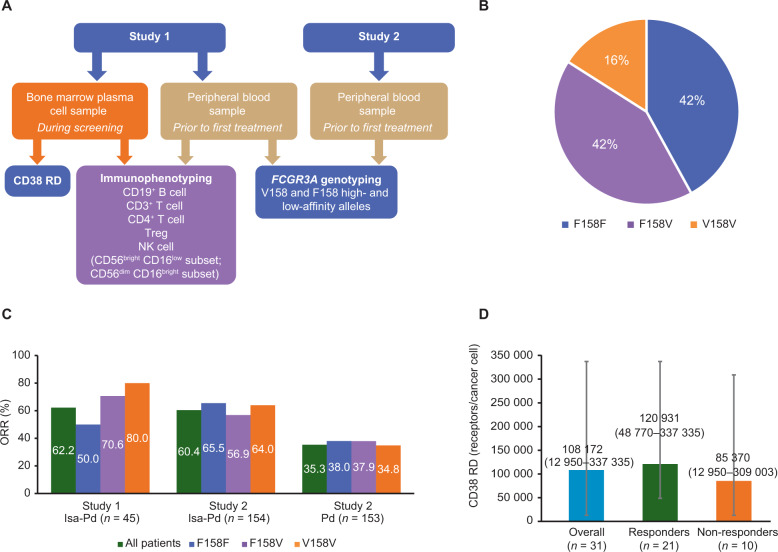
Overview of the biomarker analyses for Study 1 and Study 2. **A** Biomarker analyses for Study 1 and Study 2. Baseline peripheral blood samples were taken prior to first treatment in both studies. In Study 1, an additional bone marrow plasma cell sample was taken during screening. CD38 receptor density (RD) was determined in bone marrow plasma cell samples. Immunophenotyping was performed in blood and bone marrow samples, which were analyzed by multiparametric flow cytometry based on the expression of cell surface markers. Immune cell populations including B cell (CD19^+^ Bcell), T cell (CD3^+^ T cell, CD4^+^ cell, regulatory T cells[Treg]), and natural killer (NK) cells were characterized. Blood samples from both studies were analyzed for germline *FCGR3A* genotyping (V158 high-affinity and F158 low-affinity alleles). **B** *FCGR3A* genotype frequency in Study 1 and Study 2. Distribution of the F158V single-nucleotide polymorphism of *FCGR3A* gene in baseline peripheral blood samples taken prior to first treatment in both studies. **C** ORR by *FCGR3A* genotype in Study 1 and Study 2. *FCGR3A* genotypes were available for 44/45 patients in Study 1, 138/154 patients in the Isa-Pd arm of Study 2, and 139/153 patients in the Pd arm of Study 2. **D** CD38 Receptor density in Study 1. Median CD38 receptor density (RD) for responders and non-responders in Study 1. Evaluable results were available for 31 patients. *d* dexamethasone, *FCGR3A* Fc immunoglobulin receptor, *Isa* isatuximab, *ORR* overall response rate, *P* pomalidomide.

Statistical analyses for Study 1 and Study 2 are detailed elsewhere^6,10^. Progression-free survival (PFS) and overall survival (OS) were analyzed by the Kaplan–Meier method. Other secondary endpoints were summarized using descriptive statistics. In the current analysis, *P*-values between groups were calculated using Wilcoxon rank-sum test.

*FCGR3A* genotypes were available for 44/45 patients in Study 1, 138/154 patients in the Isa-Pd arm, and 139/153 patients in the Pd arm of Study 2. Across both studies, the frequency of the F158F and F158V genotypes of the *FCGR3A* gene was equal at 42% each, whereas the V158V genotype occurred at 16% frequency (Fig. 1B). In addition, across all patients treated with Isa-Pd, the ORR was similar between the two studies (62.2% [28/45] in Study 1 vs. 60.4% [93/154] in Study 2), and responses were observed for all *FCGR3A* genotypes (Fig. 1C). In Study 1, the ORR varied by genotype; the highest ORR (80%; 4/5 patients) was observed with the V158V population, while the ORR was 70.6% (12/17 patients) with the F158V variant, and 50% (11/22 patients) with the F158F variant. In contrast to Study 1, the ORR was similar for all *FCGR3A* genotypes for patients treated with Isa-Pd (range 56.9–65.5%) from the larger phase-3 Study 2. Importantly, treatment with Isa-Pd demonstrated improved ORR over that observed with Pd treatment, not only in all patients (60.4% vs. 35.3%), but also across all three genotypes.

In Study 2, a significant PFS benefit with Isa-Pd vs. Pd was observed in the overall population (HR 0.596, 95% CI 0.436–0.814). Consistent with this, PFS benefit with Isa-Pd was observed across all three *FCGR3A* genotypes (HR range 0.447–0.728), with the highest PFS benefit observed for the V158 variant (14.78 months vs. 4.47 months) (Table 1). However, no clear association was seen between Fc polymorphism and PFS; homozygous F158F (HR 0.561, 95% CI 0.329–0.957) and V158V (HR 0.447, 95% CI 0.190–1.048) variants have similar HRs, whereas the heterozygous F158V variant (HR 0.728, 95% CI 0.450–1.178) has a less pronounced HR.

**Table 1:** Predictive value of baseline immune biomarkers.

	Isa-Pd		Pd		
	*n*	Median PFS (months)	*n*	Median PFS (months)	HR (95% CI)
*PFS by FCGR3A genotype in Study 2*					
Overall	154	11.53	153	6.47	0.596 (0.436–0.814)
F158F	55	11.53	50	7.03	0.561 (0.329–0.957)
F158V	58	8.97	66	7.43	0.728 (0.450–1.178)
V158V	25	14.78	23	4.47	0.447 (0.190–1.048)

Immune cell population (median cells x 10^9^/l)	*P-*value
*Predictive value of baseline peripheral blood biomarkers for response to Isa-Pd in Study 1*	
CD19^+^ B cell (0.0121)	0.1827
CD3^+^ T cell (1.0061)	0.7390
CD4^+^ T cell (0.6087)	0.8176
Treg (0.0147)	0.9184
NK cells (0.0351)	0.3563
CD56^bright^ CD16^low^ NK cell (0.0245)	0.4122
CD56^dim^ CD16^bright^ NK cell (0.0102)	0.2705
*Predictive value of baseline bone marrow biomarkers for response to Isa-Pd in Study 1*	
CD19^+^ B cell (0.6200)	0.2817
CD3^+^ T cell (7.9100)	0.6446
CD4^+^ T cell (7.4811)	0.7780
Treg (0.1200)	0.1620
NK cells (1.0200)	0.9591
CD56^bright^ CD16^low^ NK cell (0.3500)	0.8275
CD56^dim^ CD16^bright^ NK cell (0.4900)	0.7389

*P*-values were determined by Wilcoxon rank-sum test.

*CI* confidence interval, *d* dexamethasone, *FCGR3A* Fc immunoglobulin receptor, *HR* hazard ratio, *Isa* isatuximab, *P* pomalidomide, *PFS* progression-free survival. *CD* cluster of differentiation, *d* dexamethasone, *Isa* isatuximab, *NK* natural killer, *P* pomalidomide, *Treg* T regulatory cell.

To test whether CD38 RD has predictive value for response to Isa-Pd treatment, we evaluated CD38 RD for responders and non-responders in Study 1. Baseline CD38 RD was measured by quantitative flow cytometry in bone marrow samples from 31 out of 45 patients. The median CD38 RD was 108,172 (range 12,950–337,335) receptors/cancer cell (Fig. 1D). There was a trend for the median CD38 RD value to be higher in patients responding to Isa-Pd (120,931 receptors/cancer cell, range [48,770–337,335], *n* = 21) vs. non-responders (85,370 receptors/cancer cell, range [12,950–309,003], *n* = 10). Responses to Isa-Pd were observed in patients across the entire spectrum of CD38 RDs with the lowest value as low as 48,770 receptors/cancer cell. In a univariate analysis the association between CD38 RD and ORR was not statistically significant (*P* = 0.2870).

Baseline levels of host immune cell subsets (CD19^+^ B cells, CD3^+^ and CD4^+^ T cells, NK cells [CD56^+^ bright CD16^+^ low and CD56^+^ dim CD16^+^ bright], and Tregs) were tested to determine if a response to isatuximab treatment could be predicted (Table 1). There was no significant difference between responders and non-responders for the immune cell markers in baseline blood samples, indicating that peripheral blood baseline levels of B-cell, T-cell, and NK-cell subsets were not predictive of response to Isa-Pd. Furthermore, percentages of host immune cell biomarkers were similar between responders and non-responders in bone marrow samples collected at baseline (Table 1).

In the current analysis, we explored the predictive value of baseline biomarkers including bone marrow plasma cell CD38 RD, *FCGR3A* genotype, and immune cell markers in blood/bone marrow for response to Isa-Pd treatment. We showed that there is no clear association between Fc polymorphism and isatuximab treatment outcome. Notably, both ORR and PFS benefit with Isa-Pd vs. Pd treatment was observed across all three *FCGR3A* genotypes (V/V, V/F, and F/F), consistent with that in the overall population. Despite the higher median CD38 RD in patients who responded to Isa-Pd (120,931 receptors/cancer cell in responders vs. 85,370 receptors/cancer cell in non-responders), there is not enough evidence to support the predictive value of CD38 RD. While some responders to Isa-Pd had RD as low as 48,770/cancer cell, non-responders had RD as high as 309,003/cancer cell. Furthermore, CD38 RD is high in MM; only 2% of the patient samples had a CD38 RD of <48,770/cancer cell, with the lowest being 12,950 receptors/cancer cell. These data indicate that CD38 RD is not a predictive biomarker and cannot be used to guide treatment decisions with Isa-Pd.

Preclinical studies showed that isatuximab eliminates CD38^+^ Tregs and restores T-cell and NK-cell–mediated antitumor immune responses^9^. In the current analysis, we explored whether baseline levels of immune cell subsets including NK cells, T cells, and B cells could predict clinical outcome with isatuximab treatment. No significant difference was observed between responders and non-responders to isatuximab treatment for the tested immune biomarkers both in peripheral blood as well as in bone marrow plasma cell samples, indicating that immunophenotyping does not predict response to isatuximab treatment.

Limitations of the current study include the small subsets of patients evaluated for some of the included analyses.

In conclusion, we did not find a significant association between tumor response and the evaluated baseline biomarkers (bone marrow plasma cell CD38 RD, peripheral blood cell *FCGR3A* genotypes, and peripheral blood or bone marrow immune cell populations) in patients with RRMM treated with Isa-Pd. Isa-Pd provides a consistent benefit to patients with RRMM, including difficult to treat subgroups. However, the results of this analysis indicate that prescreening patients for these clinical and biomarker characteristics may not be warranted for Isa-Pd.

## Data Availability

Qualified researchers can request access to patient-level data and related study documents including the clinical study report, study protocol with any amendments, blank case report forms, statistical analysis plan, and dataset specifications. Patient-level data will be anonymized, and study documents will be redacted to protect the privacy of trial participants. Further details on Sanofi’s data-sharing criteria, eligible studies, and process for requesting access are at https://www.clinicalstudydatarequest.com.

## References

[1] Rajkumar SV (2016). Multiple myeloma: 2016 update on diagnosis, risk-stratification, and management. Am. J. Hematol..

[2] Richardson PG (2018). Isatuximab plus pomalidomide/dexamethasone versus pomalidomide/dexamethasone in relapsed/refractory multiple myeloma: ICARIA Phase III study design. Future Oncol..

[3] van de Donk N, Richardson PG, Malavasi F (2018). CD38 antibodies in multiple myeloma: back to the future. Blood.

[4] Nijhof IS (2016). CD38 expression and complement inhibitors affect response and resistance to daratumumab therapy in myeloma. Blood.

[5] Deckert J (2014). SAR650984, a novel humanized CD38-targeting antibody, demonstrates potent antitumor activity in models of multiple myeloma and other CD38^+^ hematologic malignancies. Clin. Cancer Res..

[6] Mikhael J (2019). A phase 1b study of isatuximab plus pomalidomide/dexamethasone in relapsed/refractory multiple myeloma. Blood.

[7] Martin TG (2019). Therapeutic opportunities with pharmacological inhibition of CD38 with isatuximab. Cells.

[8] Atanackovic D (2020). In vivo vaccination effect in multiple myeloma patients treated with the monoclonal antibody isatuximab. Leukemia.

[9] Feng X (2017). Targeting CD38 suppresses induction and function of T regulatory cells to mitigate immunosuppression in multiple myeloma. Clin Cancer Res..

[10] Attal M (2019). Isatuximab plus pomalidomide and low-dose dexamethasone versus pomalidomide and low-dose dexamethasone in patients with relapsed and refractory multiple myeloma (ICARIA-MM): a randomised, multicentre, open-label, phase 3 study. Lancet..

[11] Mahaweni NM (2018). A comprehensive overview of FCGR3A gene variability by full-length gene sequencing including the identification of V158F polymorphism. Sci. Rep..

